# Supplementary material for: Are carbon emissions trading and green financial instruments synergistic? -Comprehensive quantitative research based on content analysis
Source: PLoS One. 2024 Mar 7;19(3):e0298601. doi: 10.1371/journal.pone.0298601 (PMC10919601; doi:10.1371/journal.pone.0298601)
Supplement: S1 Data — (ZIP) [file pone.0298601.s001.zip › Data/地级市Z_F工作报告.zip!/╔┬╬≈/▒a╝a╩╨/└┤╫╘╣1⁄2╓┌║┼í╛┬φ┐╦╩2╛▌═°í┐_2008.docx]

现在，我代表市人民政府向大会作工作报告，请予审议，并请市政协委员和其他列席人员提出意见。

　　过去五年的工作

　　市十二届人大一次会议以来，在市委的正确领导下，市政府以邓小平理论、“三个代表”重要思想为指导，以科学发展观统揽经济社会发展全局，紧紧围绕建设“四市”、创建“两个最佳”、实现“三个率先”、建设和谐奋进新宝鸡的目标，开拓创新，锐意进取，圆满完成了各项目标任务，开创了科学发展、和谐发展的新局面。

　　经济实力大幅提升。五年来，生产总值以年均13.8%的速度增长，预计2007年实现地区生产总值580亿元、财税总收入67亿元，分别比2002年增长1.32倍和1.44倍，其中地方财政收入20亿元，比2002年同口径增长1.58倍。综合实力在全国排名由2003年的81位提升为2006年的第73位。经济结构进一步优化,运行质量显著提高，三次产业比例调整为10.4:60.8:28.8。工业经济保持强劲增长，规模以上工业增加值年均增长22.3%，增速连续五年位居关中前列。节能减排工作全面开展并取得明显成效。第三产业快速发展，增加值年均增长10.4%，跻身首批“中国旅游竞争力百强城市”,扶风县荣获中国旅游强县称号。非公有制经济、县域经济不断壮大，占全市经济总量比重分别比2002年提高6.8和4.3个百分点。高新区继续保持高速增长的良好态势，预计今年生产总值130亿元，主要经济指标增速连续4年位居全国54个国家级高新区前列，已成为全市经济发展的重要增长极。

　　“三农”工作成效显著。农业产业结构进一步优化，畜牧业产值占农业总产值的比重比2002年提高3.5个百分点。农业产业化步伐明显加快，销售收入过千万元的龙头企业达到86户。新型农业科技推广服务体系基本建立，24个农业科技专家大院带动作用明显。支农政策使广大农民得到更多实惠，农业税、特产税全部取消，粮食直补政策全面落实，农机、良种等补贴政策受益面不断扩大。农民收入持续增长，预计2007年农民人均纯收入2773元，比2002年增加1087元。新农村建设扎实推进，全市94%的乡镇通上了油路，80%的行政村通上了沥青（水泥）路；建成农村供水工程1195处，解决了75.8万人的饮水困难，农村面貌发生了显著变化。“突破西山”和凤县率先脱贫取得重大进展，贫困地区群众的生产生活条件明显改善，全市29.6万农村贫困人口实现了脱贫。

　　改革开放深入推进。国有企业改革走在全省前列，47户企业改制重组、9户企业破产、21户省属企业接收工作圆满完成，有效处置企业金融债务16亿元。农村综合改革逐步深化，粮食流通体制改革全面完成。对外开放步伐加快，成功举办了森博会、五届“两节一会”、两届全球华人省亲祭祖大会，积极参加国内外各类经贸活动，宝鸡的知名度和影响力不断扩大。招商引资效果显著，五年累计合同引资1082.9亿元，实际到位资金442.9亿元，法国施耐德等4户世界500强及青岛啤酒、杭州叉车、徐矿集团等一批国内外著名企业落户宝鸡。预计2007年完成进出口总额6.8亿美元，比2002年增长8.3倍，进出口总额跃居全省第二位。

　　项目建设成果丰硕。五年来,全市共实施重点建设项目319个，累计完成全社会固定资产投资969.8亿元，年均增长27.4%。产业项目建设实现重大突破，冀东扶风水泥、东岭集团锌冶炼、金德管业、宝鸡机床技改、蒙牛乳业、华龙日清、千阳陶瓷工业园等一批产业项目建成投产，宝钛工业园、长岭纺电工业园、中小企业园、宝烟易地改造等项目进展顺利。基础设施日趋完善，宝兰铁路复线、姜眉公路、关中公路环线等建成通车，人畜饮水、大中型灌区更新改造、城乡电网改造全面完成，宝天高速、宝平路改造等项目建设进展顺利。广播电视“村村通”、有线电视网络不断延伸，通讯、互联网覆盖面逐年扩大。

　　城市化步伐明显加快。城市空间进一步拓展，宝鸡县撤县设区（陈仓区）顺利完成，城市建成区面积达到67.5平方公里，比2002年增加 23.9平方公里。陈仓大道、渭滨大道、高新大道、滨河大道、福潭大桥、卧龙大桥等建成通车，“四横十纵”的城市立体交通骨架初步形成。市行政中心建设即将竣工，供热中心、天然气加压站、垃圾处理场、污水处理厂建成投用。城市管理综合执法试点工作为全国创造了经验，城市精细化管理成效明显，城市小道路改造全面完成，旧城改造、城中村改造和北坡移民搬迁步伐加快。渭河市区段、铁路沿线、引渭渠综合治理效果显著。城市生态环境明显改善，金渭湖、金陵河、清姜河橡胶坝全部建成，市区公园免费开放，南山北坡绿化进展顺利，绿色宝鸡建设成效显著，顺利通过国家绿化模范城市验收。大力实施“突破蔡家坡”战略，关中西部城镇群加速崛起。

　　社会事业全面发展。科技创新能力不断增强，成为全国技术创新示范城市。义务教育进一步加强，“两免一补”政策全面落实，宝鸡文理学院、宝鸡职业技术学院和宝鸡中学新校区建成投用。文化事业和文化产业快速发展，文化艺术中心、妇女儿童中心等“十大文化工程”建设进展顺利。农村三级医疗卫生网络逐步完善，市县疾控中心、急救中心和72个社区卫生服务机构建成投用，成功处置了“非典”、禽流感等突发事件，公共卫生保障能力进一步增强。在全省率先实现省级卫生县城全覆盖，千阳、凤翔、凤县建成国家卫生县城。人口自然增长率控制在5‰以内。全民健身和竞技体育取得新成绩。社会治安综合治理继续深化，“平安宝鸡”创建成效明显。信访突出问题得到集中解决，安全生产不断加强，全市社会大局和谐稳定。

　　人民生活持续改善。民生问题得到各级政府的普遍重视和关注，养老保险、医疗保险、社会救助基本实现了制度设计上的全面覆盖，各项社会保险参保人数达到166.5万人。新型农村合作医疗、城镇居民医疗保险、新型农村养老保险、城乡低保、养老事业等工作走在了全省前列，社会保障水平在全国百强城市中列46位。创业促就业的新机制基本形成,五年来全市城镇累计新增就业22.8万人，帮助10.6万名下岗失业人员实现了再就业。2007年，预计城镇居民人均可支配收入达到10980元，比2002年增加4494元。

　　人事、监察、国土、审计、统计、档案、物价、地志、人防、地震、气象、侨务以及妇女儿童、民族宗教、扶残助残和台湾事务等各项工作都取得了新的成绩。

　　各位代表，过去五年，我市先后在西北地区率先建成了国家卫生城市、环保模范城市、园林城市和全国绿化模范城市，成为中西部地区唯一的“中国品牌经济城市”，实现了全国双拥模范城“六连冠”，荣获中华环境奖、全国社会治安综合治理优秀城市和全省唯一的省级文明城市等多项殊荣。五年成就的取得，是市委驾驭全局、正确领导的结果，是市人大、市政协鼎力支持、共谋发展的结果，是全市人民共同奋斗、辛勤劳动的结果。在此，我代表市政府，向全市广大工人、农民、知识分子和干部，向给予政府工作大力支持和监督的人大代表、政协委员、各民主党派、工商联、各人民团体和社会各界人士，向关心和支持政府工作的人民解放军、武警驻宝部队全体官兵和公安干警表示崇高的敬意和诚挚的感谢！

　　各位代表，过去的五年，是我市经济快速发展、质量和效益显著提高的五年，是各项改革深入推进，对外开放继续深化的五年，是社会事业全面进步、人民群众得到更多实惠的五年，是生态环境明显改善，大城市面貌发生深刻变化的五年，是政府自身建设不断加强，社会保持和谐稳定的五年。五年来的工作实践，为宝鸡今后发展积累了丰富的经验，奠定了坚实的基础。五年来，我们深深体会到，贯彻落实科学发展观，必须始终坚持把加快发展作为第一要务，着力推动经济和社会的科学发展、协调发展；始终坚持把改善民生作为第一宗旨，着力强化为民、利民、富民的执政理念；始终坚持把改革创新作为第一动力，着力破解发展难题；始终坚持把项目带动作为第一战略，着力引进大项目促进大发展；始终坚持把和谐稳定作为第一责任，着力解决人民最关心、最直接、最现实的利益问题。

　　回顾五年来的工作，我们也清醒地看到，在前进的道路上还存在着不少困难和问题。主要是：经济发展速度还不够快，运行质量和效益还不够高，发展方式有待进一步转变；城乡和区域发展还不够协调，县域经济总量还不够大，新农村建设任务繁重，农民收入增长缓慢；公共服务均等化水平亟待提高，社会建设任重道远。对于这些问题，我们必须高度重视，采取有力措施认真加以解决。我们坚信，只要我们与时俱进，开拓创新，励精图治，就一定能够开创宝鸡发展新篇章，再铸宝鸡建设新辉煌！

　　今后五年的奋斗目标和重点任务

　　党的十七大为我国全面建设小康社会描绘了宏伟蓝图，市十次党代会提出了实现“三个率先”、建设和谐奋进新宝鸡的奋斗目标，这些都将成为今后政府工作的重要指导方针。今后五年，是我市经济社会发展的关键时期。从国际形势看，世界格局正处于向多极化过渡的重要时期，经济全球化趋势继续在曲折中发展，主要发展中国家均呈现出强劲的增长势头。从国内形势看，国家将继续实行稳健偏紧的宏观调控政策，但由于经济增长的强大惯性，我国经济仍将运行在这一轮经济周期的上升期；西部大开发继续深入推进，关中经济带将迅速崛起；以改善和保障民生为重点的社会建设，将成为国家长期的政策走向。从我市发展看，通过近年来的努力，综合竞争力逐步提高，发展的协调性进一步增强。可以说，我们面临的机遇前所未有，挑战也前所未有，但总体上看机遇大于挑战。站在新的历史起点上，我们既不能安于现状，固步自封，也不能脱离实际，急于求成，必须始终保持清醒的头脑，进一步解放思想，求真务实，坚定信心，乘势而上，努力开创经济社会又好又快发展的新局面。

　　今后五年，政府工作的总体要求是：以科学发展观为指导，全面贯彻落实党的十七大精神，立足“三个率先”，转变发展方式，优化经济结构，坚持走新型工业化道路，提高新农村建设水平，加快推进城市化进程，加强社会建设，着力改善民生，繁荣文化事业，建设生态文明，努力构建城乡经济繁荣、人民安居乐业的和谐奋进新宝鸡。

　　 今后五年全市经济社会发展的奋斗目标是：

　　 ——经济实力跃上新台阶。生产总值年均增长14%以上，到2012年力争达到1200亿元，人均GDP突破3万元；财税总收入力争达到150亿元，其中地方财政收入55亿元，工业总产值达到1900亿元，产业结构得到优化，综合实力显著增强。

　　 ——社会建设取得新发展。社会事业全面进步，公共服务更加公平，城乡居民的教育、文化、卫生、住房等条件明显改善；民生得到切实保障，就业更加充分，覆盖城乡居民的社会保障体系进一步完善。农村贫困人口减少25万，城镇居民人均可支配收入和农民人均纯收入力争达到19700元、4800元，城乡居民幸福感和满意度不断提高。

　　 ——大城市建设迈出新步伐。城市载体服务功能进一步完善，带动和辐射作用更加突出，在西部率先建成现代化大城市。城市管理水平显著提高，大中小城镇协调发展，城镇人口比重明显增加，城市化率达到50% 。

　　 ——和谐宝鸡建设谱写新篇章。生态文明观念牢固树立，主要污染物排放得到有效控制，环境质量明显提高。民主更加完善，法制更加完备。社会治安防控体系进一步健全，社会大局安全稳定。

　　根据上述总体要求和奋斗目标，今后五年重点抓好六个方面的工作：

　　一是以新型工业化为主导，推动工业经济快速增长。我市正处于由工业化中期阶段向高级阶段迈进的关键时期。要进一步强化工业的支柱地位，坚定不移地走新型工业化道路，转变经济发展方式，推动工业经济结构优化升级。积极实施大企业大集团、项目带动和自主创新三大战略，加快四大基地、五大产业集群建设，大力推进信息化与工业化融合，突出抓好国企改革和招商引资，继续保持工业经济快速发展的良好态势。力争在五年内，全市培育形成年销售收入过百亿元的企业5户、过10亿元的企业30户。加快非公经济发展，抓好“两区多园”建设，强力推进县域工业化。

　　二是以新农村建设为抓手，促进农村全面繁荣。建设社会主义新农村是一项长期的重要工作。要以新农村建设统揽三农工作，把发展现代农业、繁荣农村经济作为首要任务。坚持工业反哺农业、城市支持农村和多予少取放活的方针，逐步建立以工促农、以城带乡的长效机制，努力构建城乡经济社会发展一体化新格局。积极推进特色农业现代化，大力发展一村一品，培育农业产业化龙头企业，健全农业社会化服务体系，推动农业科技进步，加强农村基础设施建设，改善农村生产生活条件，促进农业稳定发展、农民持续增收。培育有文化、懂技术、会经营的新型农民，扩大农村劳动力转移规模。积极实施“突破西山”战略，提高全市扶贫开发水平，基本实现农村贫困人口脱贫目标。

　　三是以建设现代化大城市为目标，加快城镇化进程。加快城镇化进程是建设现代化大城市的必然要求。坚持走新型城市化道路，按照统筹城乡、布局合理、节约土地、功能完善、以大带小的原则，全面推进大城市建设。加快东扩南移北上步伐，拓展城市发展空间，加强基础设施建设，进一步提升城市载体服务功能。加大城市管理工作力度，建立健全长效机制，提高城市精细化管理水平。全面推进县城、重点镇建设，加快构建关中西部城镇群。深化创建活动，突出生态环保、人文特色两大优势，做好“山水”文章，培育城市精神，提升城市品位。到2010年，建成“国家森林城市”、“宜居城市”，力争摘取“联合国人居环境奖”。

　　四是以弘扬先进文化为主题，大力繁荣文化事业。先进文化是经济社会发展的重要智力支持。要大力繁荣文化事业，不断深化文化体制改革，积极扶持公益性文化，着力发展文化产业，增强文化发展活力。进一步加大投入力度，加强农村和城市社区文化设施建设。建设和谐文化和社会主义核心价值体系，培育文明风尚。有效整合文化旅游资源，加快建设文化旅游名市。集中力量培育一批有影响力的文化作品，兴办一批有代表性的文化设施，开展有针对性的文化服务，全面提升和谐文化引领发展、服务发展的能力，让全市人民共享先进文化的发展成果。

　　五是以改善民生为重点，着力推进社会建设。社会建设是构建和谐社会的基础性工程。坚持教育优先发展，切实提高农村义务教育水平，加快普及高中阶段教育，大力发展职业教育，扩大优质教育资源规模，鼓励和规范社会力量兴办教育。进一步完善农村三级卫生服务网络和城市社区卫生服务体系，建设覆盖城乡居民的公共卫生服务、医疗服务、医疗保障和药品供应保障体系，提高全民健康水平。全面落实积极的就业政策，加强就业服务体系建设，改善就业环境，鼓励全民创业。不断完善基本养老、基本医疗、最低生活保障制度，加快建立社会福利、社会救助体系，全面提高保障水平。强化安全生产管理，加强社会治安综合治理，维护社会安定团结。

　　六是以建设生态文明为核心，增强可持续发展能力。生态文明是科学发展观的重要内涵。以建设国家生态示范市为目标，深入开展生态示范县区创建活动。加快南山北坡、铁路公路沿线绿化和荒山造林步伐，加大渭河两岸生态治理力度，用3—5年时间，构筑起绿色宝鸡框架。高度重视节能减排工作，积极推行资源节约和循环利用，加大耕地保护和复垦力度，推广中水利用，发展环保节能产业，倡导绿色消费，努力建设资源节约型、环境友好型社会。

　　新起点承载着新希望、孕育着新生机、迸发着新活力，只要我们把人民的期盼变成不断前进的动力，把历史的使命化为真抓实干的行动，创造精彩，拥抱辉煌，“三个率先”的奋斗目标就一定能够实现。

　　2008年的主要工作

　　各位代表，2008年是贯彻落实党的十七大精神的重要一年，也是新一届政府展示新形象、谋划新发展、体现新作为的关键一年。市政府将始终坚持经济建设不放松，进一步增强发展的协调性，不断满足人民群众日益增长的物质需求；始终坚持全面推进社会建设，切实保障和改善民生，不断提高公共服务水平；始终坚持城市化带动战略，全力抓好大城市建设和管理，不断创造良好的人居生活环境；始终坚持转变政府职能，努力建设服务型政府, 不断推进管理和服务方式的创新。

　　2008年，全市经济社会发展的主要预期目标是：生产总值增长14％；财税总收入和地方财政收入均增长20％；全社会固定资产投资增长30％；居民消费价格总水平控制在4％左右；城市居民人均可支配收入和农民人均纯收入分别增长13.5％、13％；万元生产总值能耗和主要污染物排放总量均降低4％；人口自然增长率控制在6‰以内；新增就业4.2万人，城镇登记失业率控制在4%以内。

　　（一）加快推进产业结构优化升级，努力实现经济又好又快发展

　　科学发展观的第一要义是发展。经济发展是政治文明、文化繁荣、社会进步的物质基础。要把推动经济发展作为政府工作的中心任务，把握发展规律，创新发展理念，转变发展方式，破解发展难题，提高发展质量，为实现“三个率先”宏伟目标奠定更加坚实的基础。

　　发展壮大工业经济实力。加快推进四大产业基地建设，大力发展五大产业集群，积极实施大企业大集团战略。年内确保宝鸡机床集团数控机床扩建、冀东海德堡水泥生产线、陕西有色金属矿业公司铅冶炼等项目建成投产；加快宝钛工业园、宝烟易地技改、长岭纺电工业园、城市轨道电气化、合力叉车扩能技改等项目建设进度，力争年内建成部分生产线；开工建设大唐热电、宝二电二期、石油机械异地扩建、秦川铸造工业园、徐矿集团煤甲醇、石油钢管连续管及套管、宝光真空断路器、麟北煤田郭家河矿井、东岭电解锌等产业项目，确保完成年度投资计划。年内全市规模以上工业增加值达到288亿元，增长25％；支持大企业大集团快速发展，宝钛集团、宝石机械等10户骨干企业产值占到全市工业总产值的45％。加快建设企业技术创新体系，支持骨干企业建立研发中心，不断提高自主创新能力。大力发展品牌经济，全力支持西凤酒、宝桥等企业创建中国名牌产品。加大信息技术推广应用力度，加速运用信息技术改造提升传统工业，不断提高工业信息化水平。切实加强金融生态环境建设，健全信用担保体系，争创西部金融生态城市。加快发展非公经济，年内非公有制经济总量占全市经济总量的比重达到46.6%。继续办好“东部行”、“智慧之光”和体保健等活动，进一步加强企业家队伍建设。集中精力打好国企改革攻坚战，切实抓好制药机械厂、新秦造纸厂等企业的公司制改造，积极推进宝鸡氮肥厂、宝鸡酒精厂等14户企业破产工作，年内基本完成国企改革任务。重点抓好红旗民爆、宝成航空仪表等企业的上市工作，力争年内有1－2户企业成功上市。完成市属企办子校移交工作。大力发展循环经济，切实加大节能减排工作力度，加快高耗能行业和企业的技术改造，全面推行制造企业清洁生产。

　　大力发展园区经济。进一步完善园区管理体制，创新内部运行机制，使“两区多园”成为集聚各种生产要素、产业特色鲜明的工业发展区，成为全市经济发展的重要增长极。市上年内将投资1亿元以上，支持园区加快发展和机制创新。进一步加快高新区发展，完善高新二期、加快高新三期基础设施建设，完成供热站、科贸大厦、高新小学、大型购物中心、高新区渭河生态公园等项目，开工建设高新大道三期、高新中学、高新幼儿园、高新医院等项目，完善载体服务功能；充分发挥高新区在产业发展中的聚集和带动作用，瞄准新材料、先进制造业、电子信息、现代食品、生物医药等重点产业，大力引进关联度高、辐射和带动性强的龙头型、基地型、旗舰型大项目，确保有40个工业项目落地，年内生产总值达到180亿元，增长40％以上，使高新区发展速度继续在全国保持领先。加大突破蔡家坡力度，加快供排水、天然气、供热管网新建及改造、道路改造绿化等项目建设进度，全面改善基础设施条件；立足现有产业优势，进一步培育壮大汽车制造及零部件加工、建筑建材、食品加工等优势产业，改造提升纺织、服装等传统产业，年内生产总值达到40亿元，增长30％以上。鼓励和支持县区发展园区经济，加大陈仓物流园、凤翔煤化工工业园、千阳陶瓷工业园、凤州科技工业园和岐山建材工业园建设力度，继续支持凤翔、扶风、眉县、金台、渭滨、陈仓等工业园区建设，大力整治园区环境，改善园区形象，加快园区产业发展步伐。年内工业园区生产总值达到65亿元，增长30％以上。

　　扎实推进新农村建设。坚持不懈地调整农村经济结构，围绕畜粮果菜药等主导产业，积极实施“百村示范、千村推进”工程，构建“一村一品”、“多村一品”、“一县一业”的板块式特色经济发展格局。建成10个千头现代化奶牛场，促进标准化奶牛示范小区发展，抓好秦川牛、瘦肉型猪、布尔羊、莎能奶山羊等优质畜产品生产基地建设，做好重大动物疫病防控工作，加快畜牧业发展步伐。大力推进农业产业化经营，积极支持爱姆生物、秦宝牧业等农产品加工企业发展壮大，确保2户企业进入国家级龙头企业行列，5户企业进入全国同行业10强。抓好15个市级农民专业合作社示范点建设。大力发展现代农业，健全农技推广网络，启动实施农业科技示范村、“农业科技人员技能提升”和“百万农民培训”工程，加强专家大院建设，重点建设20个农业科技示范园和400个农村信息站。落实农业机械补贴政策，提升现代农业装备水平。新增灌溉面积2万亩，发展节水灌溉10万亩，建设基本农田2万亩，治理水土流失200平方公里，积极实施农业综合开发项目，进一步提高农业综合生产能力。以农村基础设施建设为重点，抓好100个新农村重点村建设，新修农村道路1500公里，解决10万人饮水困难，发展农村有线电视2万户，新建沼气池1.2万个。开展农村生态家园创建工作，改善农村生产生活条件。以突破西山为重点，继续打好扶贫攻坚战，抓好凤县率先脱贫示范工作，全市启动65个扶贫重点村建设，完成5000名农村贫困人口扶贫移民搬迁，实现9万农村贫困人口脱贫目标。

　　加快发展第三产业。大力发展旅游业，改善基础设施条件，完善服务功能，优化服务环境。加大太白山、关山草原、紫柏山等主要景区开发力度，法门寺合十舍利塔建成开放。加快青铜器博物院、石鼓山公园建设步伐，大力发展乡村旅游，全力抓好西岐民俗园、西部兰花园、关中风情园等一批乡村旅游示范点建设。精心策划包装旅游项目，加大旅游宣传推介，积极引进外来资金参与旅游产业开发。加快周公庙、钓鱼台等旅游景区管理体制改革，形成统一规划、有序开发的格局。全年实现旅游综合收入63.9亿元，增长17.2%。大力发展物流产业，加快陈仓物流园区会展中心建设步伐，启动园区商务酒店、信息大厦、供排水、电力、热力、通信、绿化等配套设施建设，完善园区主次干道，年内实现“七通一平”，积极引进大型物流企业入区发展。完善城市商贸网络体系，加快国际万象商业广场、嘉隆国际商城、鑫旺国际商城等重点商业网点建设，积极实施电子商务、双百市场、外经贸促进等六大工程，抓好商贸市场建设，构筑现代流通体系。加快推进交通运输、邮电通信、贸易餐饮等传统服务业升级改造，支持银行、保险、证券业等现代服务业持续发展，大力发展科技、信息、咨询、会计、审计、法律等新兴服务业。积极扩大住房、汽车、文化、休闲等消费，培育和拓展保健、假日、养老服务等新的消费增长点。年内完成社会消费品零售总额218亿元，增长16.5％。

　　全力抓好重点项目建设。建立多元化投融资机制，积极争取中省预算内资金、专项资金和民间资金投入我市经济和社会事业发展，确保投资保持较快增长，全年完成全社会固定资产投资400亿元。全面落实项目法人责任制，强化项目管理，保证工程质量，加快重点项目建设进度。抓好宝天高速宝鸡段、西宝中线虢镇至绛帐段改造等交通项目，加快引红济石、引石过渭等水利工程建设，完成宝鸡750千伏和直流500千伏输变电、硖石330千伏输变电站、城乡电网改扩建等电力项目年度投资计划，开工建设陇县千河流域、眉县横渠等土地开发整理项目。年内70个重点项目完成投资190亿元。做好重大项目前期工作，围绕国家产业政策和投资方向，积极谋划争取一批后续项目，确保项目接替有序。

　　积极扩大对外开放。要把国家“两防止”和从紧的货币政策与宝鸡加快发展有机结合起来，进一步加大招商引资力度，增强经济发展后劲。突出企业和园区招商引资的主体地位，围绕四大产业基地、五大产业集群进行招商，着力引进辐射能力强、就业空间大的重大产业项目，实现由引进项目、资金向延长产业链条的转变。努力改善投资环境，完善招商引资服务体系，实行一个项目、一套班子、一项制度，加强跟踪协调和服务，提高项目履约率和资金到位率。全力办好第二十届西交会和第八届“两节两会”，确保年内实现合同引资额500亿元，到位资金150亿元。进一步扩大对外开放，积极发展外向型经济，重视引进先进技术、先进管理和海外智力，大力开拓国际市场，扩大石油钢管、石油钻采设备、钛制品、机床工具等优势产品的出口规模，年内实现外贸进出口总额6.8亿美元。

　　(二)加快推进社会建设，努力构建和谐社会

　　社会建设与人民幸福安康息息相关。要把社会建设作为政府执政为民的重要内容，加快发展社会事业，进一步完善保障制度，着力改善民生，加强社会管理，促进社会公平正义，解决好直接关系人民群众根本利益的现实问题，使全市人民学有所教、劳有所得、病有所医、老有所养、住有所居。

　　构建民生财政体系。民生财政是改革发展的必然结果。进一步加强依法治税，保持财税收入持续快速增长。加强政府非税收入管理，增强政府调控财力的能力。进一步规范财政分配关系，全面实施政府采购、国库集中收付和“收支两条线”等预算管理制度。积极调整和优化财政支出结构，推进基本公共服务均等化，把更多财政资金投向公共服务领域，不断加大对重点支出项目的保障力度，把新增财力的重点向新农村建设倾斜，向社会事业发展的薄弱环节倾斜，向困难地区倾斜，不断改善人民群众的生产生活条件，满足人们对公共产品的需求，让广大人民群众共享改革发展成果、同沐公共财政阳光。

　　推动和谐文化建设。文化是民族的血脉和灵魂。加快建设公共文化服务体系，积极实施文化信息资源共享等文化惠民工程。加快文化艺术中心、广播电视中心、图书馆改造等项目建设，积极开展文艺调演、送戏送书下乡等活动，丰富广大农村群众的精神生活。大力培养文化艺术专业人才，创作反映时代精神、群众喜闻乐见的优秀文化产品，满足社会不同群体的文化需求。加强非物质文化遗产保护，扶持民间艺术发展。围绕周秦、姜炎、青铜和佛文化四大品牌，加快文化产业基地和区域性特色文化产业群建设。积极参加2008年奥运会文艺演出、澳大利亚悉尼中国文化年等对外文化交流活动，展示宝鸡丰厚的文化底蕴。加快广电事业发展，完成 “村村通”、农村有线电视和电视数字化转换工作年度任务。深入开展各类群众性精神文明创建活动，年内确保全国文明城市创建成功。加大文物保护力度，实施秦公一号大墓墓坑、周原召陈宫殿遗址等保护利用工程。以北京奥运会举办为契机，开展全民健身活动，办好全市希望之星运动会。

　　实施教育优先发展战略。教育是民生之基。积极推进“双高普九”，实施农村义务教育提升工程，完善中小学校布局，加大寄宿制学校建设力度，取消复式教学班，优化教育结构，推动教育资源向农村、贫困地区倾斜，促进义务教育均衡发展。认真落实职业教育和技能人才培养扶持政策，加强“双师型”教师队伍和实训基地建设，支持民办职业教育发展壮大，继续加快宝鸡职业技术学院、宝鸡技术学院建设，发挥带动示范作用，促进职业教育健康快速发展。加快普及高中阶段教育，积极发展远程教育和继续教育。进一步落实义务教育经费保障机制，免除城市义务教育学杂费和农村义务教育课本费，完善城乡贫困家庭学生上学救助政策，保障经济困难家庭、进城务工人员子女平等接受义务教育。深化教育改革，加强教师队伍建设，推进素质教育，全面提高教育质量。开展全民读书活动，建设全民学习、终身学习的学习型社会。

　　健全卫生服务体系。医疗是民生之需。加强疾病防控工作，提高处置突发公共卫生事件能力。进一步深化乡镇卫生院分配制度改革，积极推行乡镇卫生院分级管理，完成64个乡镇卫生院、846个村卫生室的设备配置，完善农村卫生服务体系。积极开展社区卫生服务机构收支两条线管理试点，社区卫生服务机构全部达到规范化运作，并纳入医保定点单位。配齐优秀全科医生，提高社区卫生服务水平。全面推行药品药械、卫生耗材集中招标采购，降低药品价格，切实做到让利于民。加大监督检查力度，确保食品药品安全。建立全市统一的新型农村合作医疗信息化管理平台，积极推行市级统筹，规范定点医院诊疗行为，开展家庭账户报销凭证试点，提高家庭账户使用率。巩固提高创卫成果，确保高标准通过国家卫生城市复检。广泛开展创建卫生乡镇村活动，着力改善农村卫生环境条件。积极开展计划生育优质服务县和计划生育新农村、新家庭创建活动，改善人口结构，提高出生人口素质。

　　鼓励全民创业促就业。就业是民生之本。以贯彻落实《就业促进法》为契机，以创业扩大就业、扶持创业培养企业家为目标，通过进一步放宽政策、改善环境，努力构建平等竞争、充满活力的创业机制和全方位的创业服务体系。积极开展信用社区试点工作，建立小额担保贷款长效机制，为创业者提供便捷的服务。创建充分就业社区，扩大政府购买公益性岗位的扶持范围。大力开展“农民创家业、能人创企业”活动，积极实施人人技能工程，抓好农村富余劳动力转移就业培训工作，提高劳动力的岗位适应能力，使各类创业主体在创业中各显其能、各展其才，以创业带动就业。

　　完善社会保障体系。社保是民生之盾。继续完善政策体系，深化制度内涵。以非公经济从业人员和灵活就业人员为重点，扩大社会保险覆盖面。推行新型农村养老保险，启动城镇居民养老保险试点，完善城镇居民医疗保险，加快县区属困难企业退休人员基本医疗保险进度，推进事业单位养老保险制度改革，积极稳妥地解决农民工社会保障问题。发挥商业保险在社会保障中的补充作用。完善城乡低保制度，建立公正合理的进入、退出机制，切实做到“应保尽保、分类施保、动态管理”。健全社会医疗救助制度，适当降低社会医疗救助起付线标准，缓解困难群众“看病难”问题。开展城镇困难家庭住房需求摸底调查，完善住房保障政策；对符合规定条件的城市低保家庭发放租金补贴；加快廉租住房建设进度，增加经济适用房、廉租住房供给，年内解决1000户困难家庭的住房问题。建立慈善爱心救助基金，建成阳光大厦，营造全社会关心慈善事业、关爱残疾人的良好氛围。抓好市儿童福利院建设。制定民办福利机构发展优惠政策，加快福利事业社会化步伐。

　　加快发展养老事业。尊老敬老是社会文明进步的重要标志。积极应对人口老龄化趋势，建立惠及全体老年人的服务体系。加强老年教育网络建设，建立市、县、乡三级老年教育阵地，力争年内建成1—2所县区老年大学。积极发展民营养老机构，鼓励民间资本建设养老院和老年公寓。年内建成10个社区养老中心，发挥3999110养老呼叫服务系统的作用，为老年人提供咨询、家政、配送等服务，探索为老服务义工制度。每个县区建成1—2所规模较大、档次较高、服务设施齐全的中心敬老院，进一步提高五保户集中供养率。全面落实为老年人服务的优惠政策，健全完善老年人救助和优待制度。大力培育老年消费市场，加快养老服务社会化体系建设。发挥华厦老年大学、夕阳红总部服务老年人的主阵地作用，使其成为市区老年人老有所学、老有所乐的重要场所。市老年事业服务局要充分发挥职能作用，实现从行政管理向社会服务的转变。整合县区老年事业服务机构，建立适应老年事业发展的组织服务体系。

　　全面加强和谐社区建设。社区是社会的细胞和政权的基础。发挥驻区单位、社区民间组织、物业管理机构、专业合作经济组织在社区建设中的积极作用，实现行政管理和社区自我管理有效衔接，依法行政和居民自治的良性互动。建立社区资源共享机制，着力扶持培育各类民间组织和社区公共服务，充分发挥志愿者的作用，满足社区居民日益增长的个体化服务需求。积极开展“文明社区”、“文明楼院”、“文明家庭”以及“绿色社区”和“绿色家庭”创建活动，提升市民文明素质。加快城乡社区基础设施建设，完善社区社会保障、社会救助、社会事务等信息网络平台。加强社区环境综合治理，努力建设人与自然和谐发展的绿色社区和生态社区。

　　切实维护社会稳定。稳定是国家和人民的最高利益。要建立健全社会安全预警机制，加强社会治安综合治理，增强城乡居民的安全感。认真做好群众来信来访、人民调解工作，最大限度激发社会创造活力，最大限度增加和谐因素。落实安全生产责任制，加大安全生产执法力度，遏制重大安全事故发生。建立健全突发公共事件应急体系，提高处置突发公共事件的能力。扎实推进“平安宝鸡”创建活动，加强城乡警务工作，依法防范和打击违法犯罪活动，保障人民生命财产安全。不断扩大基层民主，完善城乡基层政权、基层自治组织、企事业单位的民主管理制度。深入开展“五五”普法宣传教育，提高全民法治意识。保障妇女儿童合法权益，抓好国防教育，加强人民防空、民兵预备役建设，巩固全国双拥模范城市建设成果。继续做好民族、宗教、外事、侨务、地震、气象、档案和地方志工作，完善社会管理，维护社会安定团结，努力形成社会和谐人人有责、和谐社会人人共享的生动局面。

　　(三)加快推进大城市建设，努力提高城市管理水平

　　城市是先进生产力和先进文化的聚集地,是政府公共服务的最大平台。要把城市建设摆在重要位置，遵循城市发展规律，加快城市基础设施建设，全面提高城市管理水平，优化人居生活环境。

　　进一步加快大城市建设步伐。以基础设施建设为重点，加快渭河以北陈仓大道沿线、秦岭北麓浅山区和蟠龙塬地区开发进程，加速人口和产业聚集，全面推进城市东扩、南移、北上。开工建设蟠龙塬东西大道、千河口跨渭河大转盘道路、秦岭北麓生态观光大道、渭河南部台塬地区道路，年内完成西宝高速辅道建设、宝虢路东段拓宽及新滨河南路、中山路、宝福路改造等建设任务。加大城市新区基础设施和公共服务设施建设力度，加快龚刘大桥、蔡阳大桥、凤凰大桥、高速客运中心、公交三场两站及污水处理、供水排水、中水回用管网等工程建设，提高配套水平和承载能力，完善城市功能。加大公共财政对城市建设的投入，多渠道、全方位筹措资金，鼓励社会各类资金参与城市基础设施建设，加快大城市建设步伐，提高辐射带动能力。

　　全面提升城市精细化管理水平。健全完善城市管理的长效机制，提高数字化城管系统运行水平，加大城市管理问题处置力度，增强城市管理的科学性。强化交通秩序和公共秩序管理，切实做到还道于民，还库于车。坚持社会开发改造、政府补贴改造、政府直接改造三管齐下，继续实施城市设施修补工程和亮化工程，加快旧城改造步伐，启动东仁堡地区和渭工路、宝十路、群众路、南关路片区等旧城改造项目。加大城中村改造力度，认真总结联盟村改造经验，完善“四个转变”政策，改善城市整体形象。加强城市管理综合执法，加大大庆路、东高新区沿街一层建筑、违章建筑、临时建筑拆除力度，以铁路沿线三期、引渭渠二期环境整治为重点，深入开展市容环境综合整治，提高城市管理水平。

　　不断加大城市生态环境治理力度。按照“一河两岸”城市景观规划，进一步加强生态环境建设，加快南山北坡绿化和生态治理，年内完成渭河、金陵河综合整治，启动千河、清水河综合整治，实施代家湾森林公园、姜城游园等生态园林工程建设，确保国家节水型城市、“中国人居环境奖”创建成功，并申报联合国人居环境奖。开工建设石鼓山渭河蓄水工程，做好蟠龙大桥东渭河及千渭之会蓄水工程的前期准备工作。加快“四沿”绿化步伐，加大荒山造林力度，扩大绿色宝鸡创建成果，争创国家森林城市。加大环境保护力度，严格城市空气质量监测，开展城市噪声综合整治，抓好水源地保护工作，强化建设项目环境监管，控制污染物排放总量，努力提升城市人居环境品位。

　　继续加强县城和小城镇建设。多方筹措资金，加强9个县城建设，完善污水处理、垃圾处理及供水、供电、供暖、通讯等基础设施。深入开展国家卫生县城、国家生态示范县区、环境优美乡镇、小康环保示范村创建活动，麟游县建成国家卫生县城，凤县、麟游、凤翔、陇县启动生态示范县创建工作，千阳县力争通过国家级生态示范县考核命名。以长青、法门、汤峪、常兴、绛帐、凤州等为重点，加快中心城镇建设，积极构筑大、中、小结合，布局合理，功能完善，协调一体的城镇群。完善就业、投资和公共服务制度，鼓励农民进城入镇就业和落户，引导农村富余劳动力向城镇集中和转移。

　　群力之所举，则无不胜；众智之所为，则无不成。宝鸡的发展离不开人民，人民的福祉更需要宝鸡的发展。只要我们尊重民意，发挥民智，凝聚民力，就一定能够创造宝鸡更加美好的明天。

　　努力建设服务型政府

　　现代政府是法治型政府，也必将是服务型政府。构建服务型政府，是生产力发展的必然要求。我们要牢固树立民本思想，进一步更新执政理念，改革管理体制，努力实现政府工作从“全能”到“有限”，从“管理”到“服务”的转变。

　　转变职能，提高效率。面对新形势新任务，各级政府要按照建立服务型政府的要求，不断改善行政管理，提高行政效能，重新审视职能定位，改变管理经济和社会事务的方式，理顺政府与企业、政府与市场、政府与社会的关系，充分发挥市场在资源配置中的基础性作用和社会自身功能在社会事务管理中的调节作用，把不属于政府职责范围的事项还权于市场调节和社会中介组织，使政府及其部门从过去繁琐的事务性工作中解脱出来，集中精力搞好公共行政，加强公共服务，逐步建立与市场经济相适应的行政管理体制。进一步改革行政管理体制，全面深入清理政府各部门行政审批事项，对不符合市场经济发展要求，能通过市场机制调节的审批项目一律予以取消，对确要保留的审批事项予以精简、规范，对部门之间重复、多头交叉的审批事项予以归并，最大限度地减少行政审批事项，规范行政行为。加快政务大厅、市民中心、中介大厦建设，确保优质便捷的公共服务体系顺畅运行。充分发挥市中介局的职能作用，加快具有中介性质的事业单位改革步伐，扶持引导金融担保、经济鉴证等中介组织和公益性行业协会加快发展。大力推进政务公开，加快建设电子政务，简化办事程序，提高办事效率，树立政府务实高效的良好形象。

　　民主决策，依法行政。政府工作人员要把学习作为一种生活习惯，努力掌握现代经济知识、科技知识、社会管理知识、法律知识，进一步提高为民服务的能力。继续完善和落实重大决策专家咨询、社会公示、社会听证和责任追究制度，大兴调查研究之风，不断改进工作方式方法，摆脱文山会海，挤出更多的时间，集中更多的精力，深入基层、深入群众、深入实际，倾听群众的意见和要求，使各项决策真正代表人民的利益，树立起新时期现代政府的良好形象。自觉接受人大及其常委会的依法监督、政协的民主监督、新闻舆论和人民群众的公开监督，虚心听取社会各界的意见、建议，认真办理人大议案、代表建议和政协委员提案，努力提高政府工作水平。政府部门都要按照法定权限和程序行使权力、履行职责，所有政府工作人员都要学会并善于依法处理经济社会事务，加强行政执法监督，促进严格执法、公正执法和文明执法，实行执法责任制和执法过错追究制，保证政府的各项工作依法进行。

　　廉洁奉公，执政为民。政府及其工作人员的一切权力来自于民，必须用之于民，让民众的根本利益得到最大保证，让民众的合理诉求得到最大满足。要增强公仆意识，正确对待和行使好手中权力。认真落实领导干部、公务员廉洁从政的各项规定，建立和完善思想教育、制度规范、监督制约、查案惩处、干部保护、廉政责任六个机制，更加注重治本，更加注重教育，更加注重制度建设，提高拒腐防变能力。深入开展反腐败斗争，依法重点查办领导干部违法违纪、执法人员贪赃枉法等案件。抓好土地批租、工程招标、产权交易等环节的监管，严格领导干部经济责任审计和“收支两条线”管理，严肃财经纪律，从源头上防止腐败行为的发生。坚持勤俭办一切事业，珍惜民资民力，精打细算，严禁铺张浪费，切实把有限的资金用在提高群众生活和增强经济发展后劲上。

　　强化责任，狠抓落实。一个服务型政府，其服务质量的优劣、服务水平的高低应由人民来评判。人民群众的利益在多大程度上得到实现，是衡量政府工作绩效的唯一尺度。各级政府必须强化责任意识,要始终坚持把抓落实作为政府工作的永恒主题，以强烈的历史使命感和时代责任感，以实现宝鸡新一轮的发展为根本职责，忠于职守，努力工作，狠抓落实，决不辜负时代与人民的重托。各级领导干部一定要增强加快发展的责任感，以时不我待、只争朝夕的精神，扑下身子，真抓实干，解决问题，推进工作，加快发展。要把着力点放在解决主要矛盾和抓好重点工作上，集中精力抓好对改革发展稳定具有决定性作用的重大问题，特别是对于事关经济社会发展全局的产业结构调整、新农村建设和社会事业等，要精心安排部署，全力抓好落实。继续加强行政效能监察，进一步健全和完善工作考评制度，制定严明的考核奖惩办法，旗帜鲜明地支持求真者，鼓励务实者，批评弄虚者，惩处误事者，切实保护和充分调动各级干部创新、创业、创优的积极性、主动性和创造性。进一步加大督办工作力度，不断完善和强化任务分解、定期汇报、督办检查、公开通报、考评奖惩等五项制度，确保工作有部署、有要求、有检查、有奖惩，促进各项工作的全面落实。

　　一个孜孜不倦追求创新的政府，一个始终坚持依法行政的政府,一个以满足社会公众需求为己任的政府,一个能够充分反映人民意愿的政府，一个廉洁奉公、执政为民的政府，必将受到人民群众的拥护和支持，必将创造出更加辉煌的业绩。

各位代表，回顾过去，我们成就辉煌、倍受鼓舞；展望未来，我们满怀豪情、充满信心。机遇不可错失，形势催人奋进。今后五年，新一届政府改革发展的任务繁重而艰巨，责任重大而光荣。让我们更加紧密地团结在以胡锦涛同志为总书记的党中央周围，深入贯彻党的十七大精神，全面落实科学发展观，在市委的坚强领导下，在市人大、市政协的鼎力支持下，紧紧依靠和团结全市人民，解放思想，求真务实，与时俱进，扎实工作，为全面建设小康社会、实现“三个率先”的宏伟目标而努力奋斗！
